# Mumps outbreak in Zimbabwe: The case for universal MMR vaccination in Africa

**DOI:** 10.1016/j.jvacx.2024.100586

**Published:** 2024-11-17

**Authors:** Phanuel Tawanda Gwinji, Grant Murewanhema, Enos Moyo, Tafadzwa Dzinamarira

**Affiliations:** a Division of Emergency Medicine, University of Cape Town, South Africa; b Unit of Obstetrics and Gynaecology, Faculty of Medicine and Health Sciences, University of Zimbabwe, Harare, Zimbabwe; c Department of Public Health Medicine, University of KwaZulu Nata, Durban, South Africa; d School of Health Systems and Public Health, University of Pretoria, Pretoria, South Africa

**Keywords:** Mumps, Vaccination, Mass vaccination, Africa

## *Dear Editor*

Cases of mumps have been recorded throughout the country of Zimbabwe. Though the total number of cases is currently unclear, Bulawayo alone is estimated to have recorded at least eight consecutive months of a mumps outbreak, with at least 240 cases of mumps reported between January and August 2023. In the recent past, mumps outbreaks have also been reported in other countries in the region, such as South Africa, Botswana, Lesotho, and Zambia. Mumps is a contagious viral infection that mostly affects children. It affects the salivary glands, causing painful swellings under the ears and parotid glands, resulting in a characteristic ‘hamster face’ appearance [5]. Other symptoms include headaches, joint pains, fatigue, loss of appetite, and fever, which often precede facial swelling. In boys, it can also result in swelling of testicles.

Mumps is usually a mild illness, but it can sometimes cause complications. These complications are more common in adults. In males, mumps can cause inflammation of the testicles (orchitis), leading to testicular atrophy. In females, it can cause inflammation of the ovaries (oophoritis) and or breast tissue (mastitis). Mumps can also cause inflammation in the pancreas, the brain (encephalitis), and deafness [1,2]. Orchitis and oophoritis are relatively common complications of mumps, while permanent sequelae such as deafness are rare [3]. While mumps orchitis rarely leads to sterility, it may contribute towards subfertility, and can also lead to oligospermia, azoospermia and asthenospermia, which are all a cause for concern in males of reproductive age. With no specific treatment for mumps, its management is focused on the relief of symptoms and the hope that no complications will occur. The disease can however be prevented through vaccination.

**Fig. 1:**
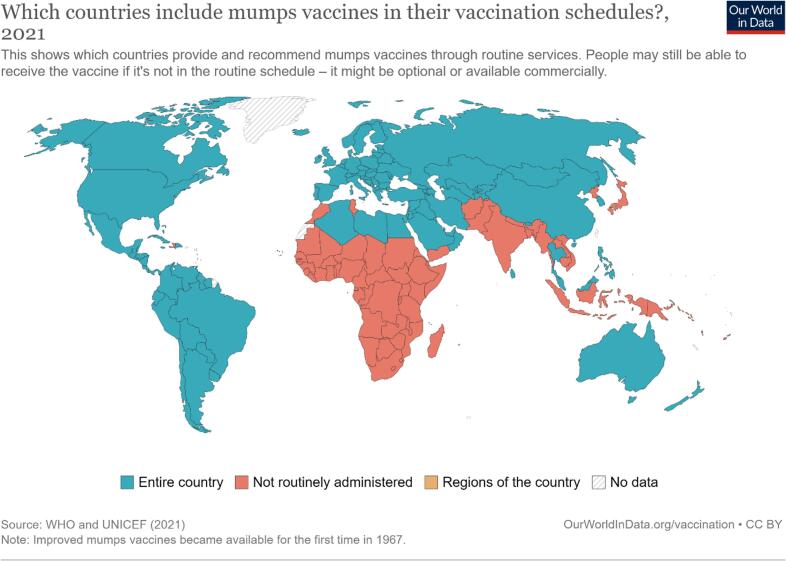
Distribution of countries that include mumps vaccines in their vaccination schedules, versus countries that do not.

Mumps vaccine is usually available as measles-mumps-rubella (MMR), or measles-mumps-rubella-varicella (MMRV). By the end of 2018, 122 countries worldwide, mostly developed countries had introduced mumps vaccine in their national immunization programs [7]. The majority of African countries do not routinely vaccinate against mumps (Fig. 1). Zimbabwe's vaccination schedule includes only the measles-rubella vaccine, given as MR1 at 9 months, and a second dose of the same (MR2) at 18 months of age. This is in contrast to the majority of countries in the global north, which give two doses of either the trivalent MMR or the MMRV variants of vaccines. Some countries also advocate for a third dose (MMR3) for high-risk populations to improve protection during outbreak settings [4]. The country's immunization schedule is based mainly on the “6 killer diseases” in Zimbabwe, which are measles, pertussis, diphtheria, tetanus, TB, and Polio. The fact that mumps is not included on the list of the “6 killer diseases” perhaps underpins the collective decision not to routinely adopt the trivalent MMR vaccine, albeit its proven efficiency and safety [4].

The CDC also reports the MMR vaccine to be very safe and effective, with the mumps component of the same being 88 % effective after two doses, and about 78 % effective with one dose [1,2]. Other studies have estimated the mumps vaccine effectiveness at around 62 % to 91 % for one dose, and 76 % to 95 % for two doses [3]. The introduction of the MMR vaccine in the United States of America resulted in a 99 % decrease in mumps cases [1,2]. Canada also saw a decline in the number of mumps cases by over 99 % since the introduction of routine two-dose MMR vaccination schedule in 1996/97 [3]. Apart from being clinically effective and safe, MMR vaccination is cost-effective. An economic analysis of a two-dose MMR vaccination program in Fiji revealed that the program was cost-effective and had a favourable budget impact when using both a taxpayer and a societal perspective [6].

Vaccines undoubtedly are a very effective public health tool available for the prevention of certain illnesses, mumps included. Though complications rarely occur, mumps affects the testis and ovaries, and we may end up having cohorts of sub-fertile adults in the next decade or two if mumps outbreaks are not controlled. As a public health measure, there is a need to conduct routine and diligent surveillance of mumps in Zimbabwe and other African countries. Consideration should also be made towards tracking mumps cases on weekly disease surveillance systems for epidemic-prone diseases which currently tracks only measles. Lastly, Zimbabwe, and other African countries, should consider adopting the trivalent MMR vaccine for the extended program on immunization (EPI).

## CRediT authorship contribution statement

**Phanuel Tawanda Gwinji:** Conceptualization, Writing – original draft. **Grant Murewanhema:** Writing – review & editing. **Enos Moyo:** Writing – review & editing. **Tafadzwa Dzinamarira:** Writing – review & editing.

## Declaration of competing interest

The authors declare that they have no known competing financial interests or personal relationships that could have appeared to influence the work reported in this paper.

## Data Availability

No data was used for the research described in the article.

## References

[1] Centers for Disease Control and Prevention (CDC) (2021). Mumps. https://www.cdc.gov/mumps/index.html.

[2] Centers for Disease Control and Prevention (CDC) (2021). Vaccines and preventable diseases. https://www.cdc.gov/vaccines/vpd/index.html.

[3] Government of Canada (2021). Mumps vaccines: Canadian Immunization Guide. https://www.canada.ca/en/public-health/services/publications/healthy-living/canadian-immunization-guide-part-4-active-vaccines/page-14-mumps-vaccine.html.

[4] Guo A., Leung J., Ayers T., Fields V.S., Safi H., Waters C. (2023). Mumps vaccine effectiveness of a 3rd dose of measles, mumps, rubella vaccine in school settings during a mumps outbreak- Arkansas, 2016-2017. Public Health in Practice.

[5] National Health Service (2021). Overview: Mumps. https://www.nhs.uk/conditions/mumps/.

[6] Oh C., Rafai E., Cho Y., Jun D., Cha S. (2022). An economic analysis of mumps vaccination in Fiji: static model simulation of routine measles-mumps-rubella (MMR) vaccination instead of current measles-rubella (MR) vaccination. Int J Environ Res Public Health.

[7] Sikhosana M.L., Kuonza L., Motaze N.V. (2020). Epidemiology of laboratory-confirmed mumps infections in South Africa, 2012-2017: a cross-sectional study. BMC Public Health.

